# Dietary butyrate glycerides modulate intestinal microbiota composition and serum metabolites in broilers

**DOI:** 10.1038/s41598-018-22565-6

**Published:** 2018-03-21

**Authors:** Xiaojian Yang, Fugui Yin, Yuhui Yang, Dion Lepp, Hai Yu, Zheng Ruan, Chengbo Yang, Yulong Yin, Yongqing Hou, Steve Leeson, Joshua Gong

**Affiliations:** 1 Guelph Research and Development Centre, Agriculture and Agri-Food Canada, Guelph, Ontario, N1G 5C9 Canada; 2 Institute of Subtropical Agriculture, Chinese Academy of Sciences, Changsha, Hunan 410125 China; 3 The Laboratory of Food Nutrition and Functional Factors, School of Food Science and Technology, Jiangnan University, Wuxi, Jiangsu 214122 China; 4 State Key Laboratory of Food Science and Technology, Nanchang University, Nanchang, Jiangxi 330047 China; 5 Department of Animal Science, University of Manitoba, Winnipeg, Manitoba R3T 2N2 Canada; 6 Hubei Key Laboratory of Animal Nutrition and Feed Science, Wuhan Polytechnic University, Wuhan, Hubei 430023 China; 7 Department of Animal Biosciences, University of Guelph, Guelph, Ontario, N1G 2W1 Canada

## Abstract

Butyrate can modulate the immune response and energy expenditure of animals and enhance intestinal health. The present study investigated changes in the intestinal microbiota composition and serum metabolites of young broilers in response to 3,000 ppm butyrate in the form of butyrate glycerides (BG) via pyrosequencing of bacterial 16S rRNA genes and nuclear magnetic resonance (NMR). The dietary treatment did not affect the alpha diversity of intestinal microbiota, but altered its composition. Thirty-nine key operational taxonomic units (OTUs) in differentiating cecal microbiota community structures between BG treated and untreated chickens were also identified. *Bifidobacterium* was, in particular, affected by the dietary treatment significantly, showing an increase in not only the abundance (approximately 3 fold, *P* ≤ 0.05) but also the species diversity. The (NMR)-based analysis revealed an increase in serum concentrations of alanine, low-density and very low-density lipoproteins, and lipids (*P* ≤ 0.05) by BG. More interestingly, the dietary treatment also boosted (*P* ≤ 0.05) serum concentrations of bacterial metabolites, including choline, glycerophosphorylcholine, dimethylamine, trimethylamine, trimethylamine-N-oxide, lactate, and succinate. In conclusion, the data suggest the modulation of intestinal microbiota and serum metabolites by BG dietary treatment and potential contribution of intestinal bacteria to lipid metabolism/energy homeostasis in broilers.

## Introduction

Butyrate, a product of bacterial fermentation of dietary carbohydrates in the digestive tract, plays a vital role in maintaining intestinal health and homeostasis as well as ameliorating the metabolic and immune status in animals. It is a preferred energy-providing substrate over glucose and glutamine for colonic epithelial cells and may count for approximately 70% of the total energy consumption of the colonocyte^1,2^. Butyrate can increase cell proliferation in the small and large intestine, and enhance the intestinal barrier by facilitating tight junction assembly^3,4^. Our recent study also indicates that butyrate can induce energy expenditure and lipid catabolism by regulating the expression of genes that have a role in the reduction of synthesis, storage, transportation, and secretion, and in the increase of oxidation of lipids and fatty acids in 3-week-old broiler chickens^5^. At the systemic circulation level, butyrate beneficially reduces the concentrations of total circulating triglycerides and cholesterol in broilers^5,6^. In addition, it has immuno-modulatory activities via histone deacetylase inhibition^7–9^. For example, butyrate induces the gene expression of antimicrobial host defense peptides, including defensins and cathelicidins in chickens^10^, as well as modulates the expression and release of anti- and pro-inflammatory cytokines in broilers^11^ and fish^12^. Nonetheless, there is limited knowledge about the effects of butyrate on the ecology of the intestinal microbiota, the whole profile of serum metabolites, and the links between them. Nuclear magnetic resonance (NMR)-based metabolomics analysis^13,14^ is a tool that can be used to assess the functionalities of nutrients via the simultaneous measurement of multiple metabolites in complex organisms, and therefore to explore how metabolic balances are disturbed by dietary nutrient interventions^15^. The advance of high throughput DNA sequencing techniques and data analyses allows comprehensive studies of the ecology of intestinal microbiota^16^. The objective of the present study was, therefore, to determine the changes in the intestinal microbiota and serum metabolites as well as their potential links in response to dietary treatment of butyrate (in the form of butyrate glycerides, BG) using a combined approach of NMR-based metabolomics analysis and metagenomics techniques including pyrosequencing of bacterial 16S rRNA genes, quantitative PCR (qPCR) assays, and NMR-based metabolomics analysis.

## Results

### Sequence analysis and quality filtering

Twelve samples per segment were used for pyrosequencing of bacterial 16S rRNA genes. After denoising, removal of chimeras, and filtering low quality sequences, the average number of DNA sequences per ileal digesta sample was 13,513 ± 2,022 with an average sequence length of 372 nucleotides. The sequences were collapsed into 489 operational taxonomic units (OTUs) at the 97% identity level. In the cecal digesta samples, the average number of sequences was 11,375 ± 2,014 per sample with an average sequence length of 383 nucleotides after the filtering. The sequences were grouped into 971 OTUs and deposited with the GenBank accession numbers MG773882 - MG774333.

### Effects of dietary butyrate glycerides on the ileal microbiota

**Figure 1:**
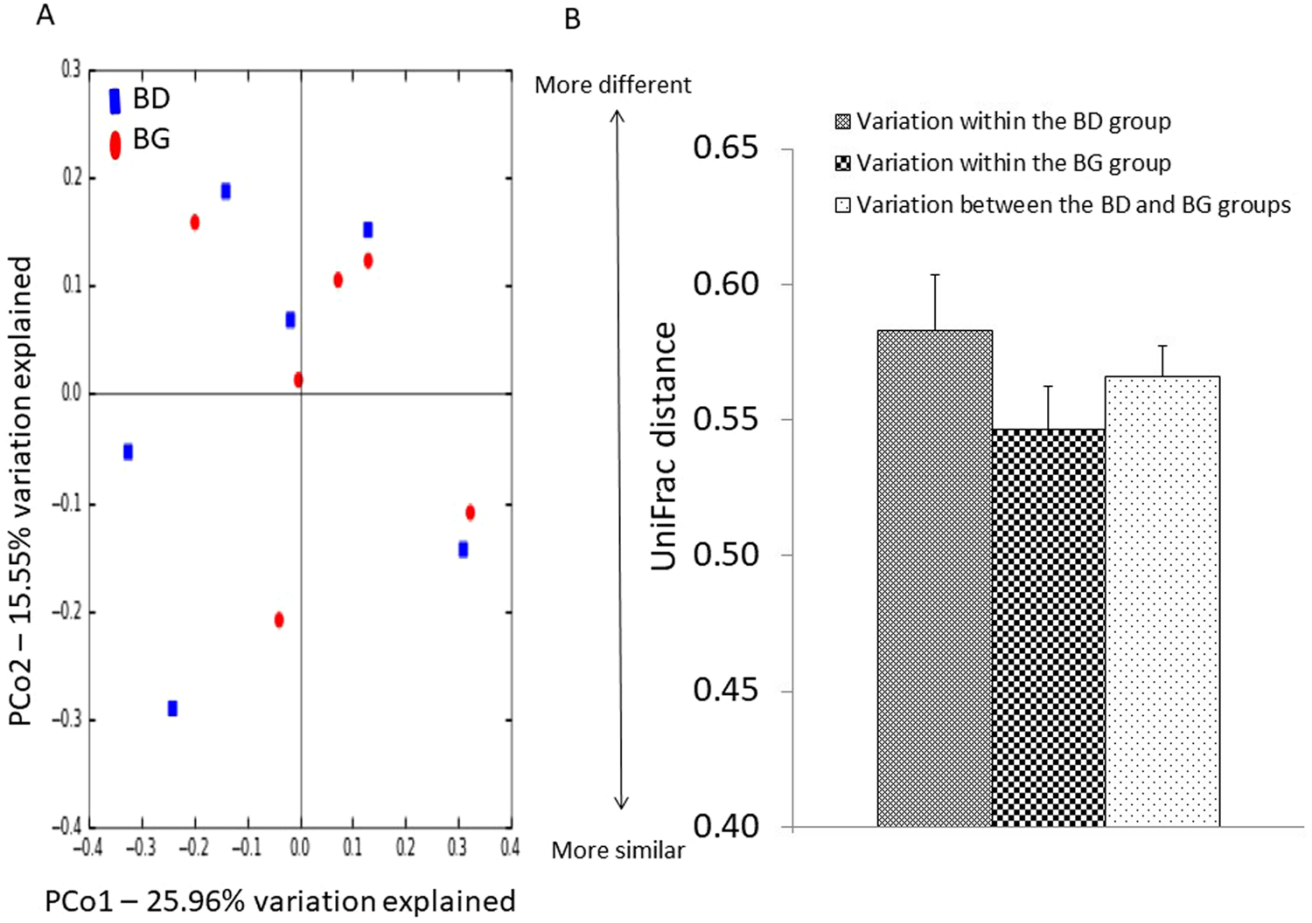
Effects of butyrate glycerides on the β-diversity of ileal microbiota. Two dimensional plots of PCoA are based on the unweighted UniFrac distance (**B**). ANOSIM is based on the unweighted UniFrac distance between microbial communities (**C**). BD: basal diet; BG: BD supplemented with butyrate glycerides.

To determine the effects of dietary treatment with butyrate glycerides on the ileal microbiota, the within-community (α) diversity was firstly assessed. The rarefaction curves including the plots of Shannon index, Chao1 index, phylogenetic distance (PD), and number of unique OTUs approached a plateau as sequence numbers increased (Fig. S1), indicating that the sequence depth was sufficient for capturing the majority of OTUs in the ileal samples. No significant treatment effects on the diversity of ileal microbiota were observed (*P* > 0.05). The similarities between pairs of microbial communities (β-diversity) were also examined using a principal coordinate analysis (PCoA) based on the unweighted UniFrac distance (Fig. 1A). No distinguishable clustering of the samples appeared to be evident between the control and treatment group (Fig. 1B). This was further confirmed by the analysis of similarities (ANOSIM) (R = 0.02, *P* = 0.42).

All OTUs with abundance ≥0.005% were taxonomically assigned with the Ribosomal Database Project (RDP) classifier at 80% confidence threshold. More than 99% of the ileal sequences were assigned to bacterial phyla. Firmicutes was the most predominant phylum (>90%) followed by Proteobacteria (4%) and Cyanobacteria (2%) (Fig. S2). At the family level, Clostridiaceae was predominant (>49%), followed by Lactobacillaceae (>21%), Enterobacteriaceae (>3%), Ruminococcaceae (>2%), and Lachnospiraceae (>1%). Over 99% of the sequences from family Clostridiaceae could not be classified further to the genus level, while *Lactobacillus* was the only genus found in family Lactobacillaceae. Analyses using the Metastats method^17^ revealed no significant difference (*P*>0.05) in the relative abundance (normalized as percentage of total number of sequences) of microbiota composition between the control and treatment group except for more than 10-fold reduction of family Bacillaceae*/*genus *Bacillus* (*P* ≤ 0.05) in BG-treated chickens.

### Effects of dietary butyrate glycerides on the cecal microbiota

**Figure 2:**
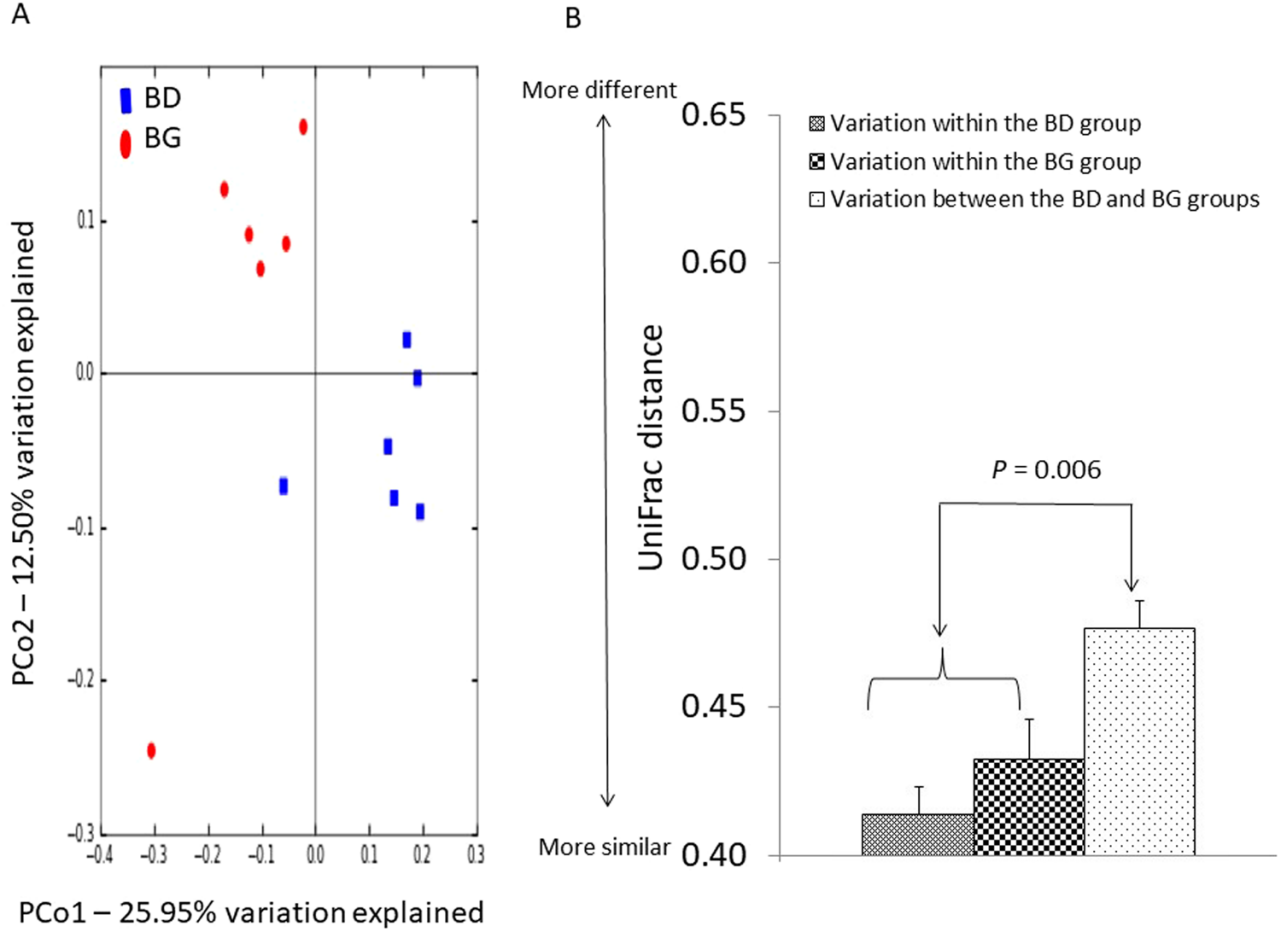
Effects of butyrate glycerides on the β-diversity of cecal microbiota. Two dimensional plots of PCoA are based on the unweighted UniFrac distance (**B**). ANOSIM is based on the unweighted UniFrac distance between microbial communities (**C**). BD: basal diet; BG: BD supplemented with butyrate glycerides.

The rarefaction curves showing the α-diversity of cecal microbiota are displayed in Fig. S1. The majority of OTUs in the cecal samples appeared to be captured. Similarly, there was no significant difference in the diversity of cecal microbiota between the control and treatment group (*P* > 0.05). The microbial community structures between the control and treatment group (β-diversity) were compared using the PCoA of unweighted UniFrac distance. Birds from the control group were distinctly separated from those in the BG group (Fig. 2A). The first and second axis of the PCoA explained 12.50% and 25.95% variation in microbial diversity, respectively. Further analysis with ANOSIM revealed a significant difference (R = 0.57, *P* = 0.006) in microbial communities of cecal microbiota between the two groups (Fig. 2B).

More than 90% of the cecal sequences were assigned to bacterial phyla. Of these sequences, more than 85% were classed to the family level, and 44% to the genus level. The cecal microbiota was dominated by Fimicutes (>76%) followed by Bacteroidetes (>11%) Tenericutes (>1%), and Proteobacteria (>0.3%), in addition to more than 6% unclassified bacteria (Fig. S2). At the family level, Ruminococcaceae was the predominant group (>40%) followed by Lachnospiraceae (>19%), Bacteroidaceae (>11%), Clostridiaceae (>4%), and Catabacteriaceae (>1%). At the genus level, *Bacteroides* accounted for more than 11% of bacterial population, followed by *Oscillospira* (>7%), *Faecalibacterium* (>7%), *Clostridium* (>3%), and *Ruminococcus* (>3%). Some sequences from order Clostridiales and families Ruminococcaceae and Lachnospiraceae could not be further classified to the genus level, which accounted for more than 7%, 23%, and 12% of the population, respectively. Analyses using the Metastats method^17^ revealed that dietary treatment with BG resulted in a decrease in the relative abundance of class Mollicutes (6-fold, *P* ≤ 0.05), and genera *Eubacterium* (2-fold, *P* ≤ 0.05), *Subdoligranulum* (20-fold, *P* ≤ 0.05), and *Holdemania* (0.03% vs. absent, *P* ≤ 0.05). On the contrary, the supplementation of BG led to a 3-fold increase (*P* *≤* 0.05) of genus *Anaerotruncus*. No differences (*P*>0.05) in relative abundance were noticed for the remaining taxonomic groups at different levels.

**Figure 3:**
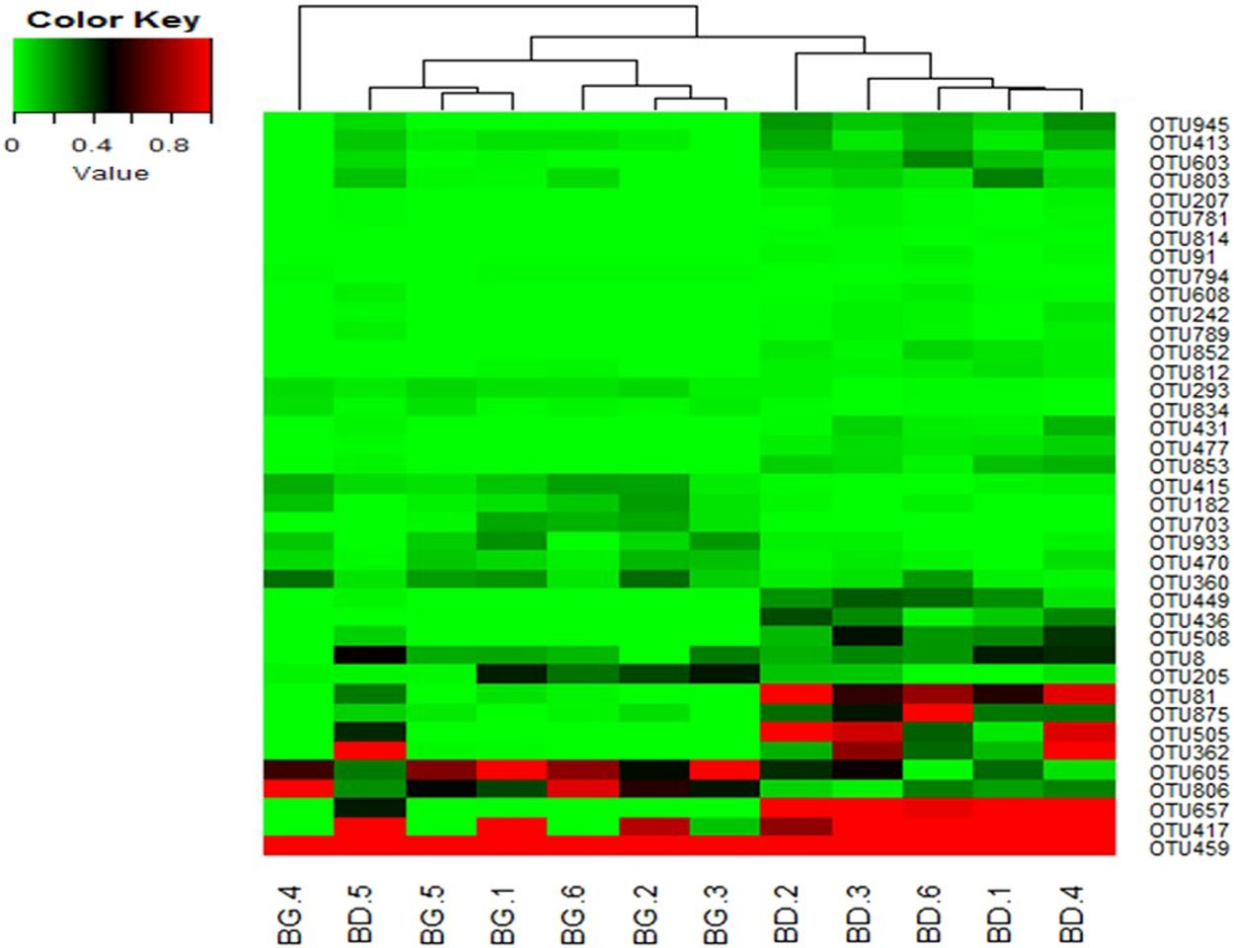
Heat map of the key OTUs identified in the cecal microbiota. The OTUs are identified by SPLS-DA as key variables for the differentiation of microbial profiles in the cecal microbiota of BG-treated and untreated (BD) groups. Percentage composition based on the 16S rRNA sequence is indicated by the color intensity. N = 6; 1, 2, 3, 4, 5, 6: number of the birds. The color key value indicates relative abundance of an OUT expressed as percentage of total number of sequences for an individual bird.

To identify key OTUs in the discrimination of microbial community structures of cecal microbiota between the control and treatment groups, sparse partial least squares discriminant analysis (SPLS-DA) was conducted. The SPLS-DA yielded a correct classification rate of 85% for the cecal samples. Thirty-nine OTUs were identified by SPLS-DA as the key variables in the differentiation of cecal microbial profiles of the two groups (Table 1). Among the 39 OTUs, the abundance of 14 OTUs was enhanced in the cecal samples from BG-treated birds, among which 8 OTUs belonged to genera *Anaerotruncus* (1), *Bacteroides* (5), *Blautia* (1), and *Lactobacillus* (1), and 6 OTUs belonged to families Lachnospiraceae (4), Ruminococcaceae (1), and Clostridiales Family XIII Incertae Sedis (1). On the contrary, the abundance of 25 OTUs was increased in the cecal samples from birds in the control group, among which 9 belonged to genera *Bacteroides* (2), *Clostridium* (1), *Oscillospira* (4), and *Subdoligranulum* (2); 12 OTUs belonged to families Ruminococcaceae (10) and Lachnospiraceae (2); 3 OTUs belonged to order Clostridiales; and 1 OTU was unclassified. The heat map of the 39 OTUs is shown in Fig. 3.

**Table 1:** Key OTUs in discriminating cecal microbial profiles between BD and BG-fed chickensa.

OTU number	Relative abundance (%) Mean ± SE (n = 6)		*P*-value	Annotation
	BD^b^	BG		
8	0.328 ± 0.024	0.121 ± 0.017	0.006	order: Clostridiales
81	0.698 ± 0.047	0.012 ± 0.004	0.0001	family: Ruminococcaceae
91	0.011 ± 0.002	0 ± 0	0.012	family: Ruminococcaceae
182	0.007 ± 0.002	0.093 ± 0.010	0.002	genus: *Lactobacillus*
205	0.051 ± 0.010	0.261 ± 0.033	0.013	family: Lachnospiraceae
207	0.013 ± 0.001	0 ± 0	0.001	genus: *Oscillospira*
242	0.019 ± 0.003	0 ± 0	0.010	order: Clostridiales
293	0.014 ± 0.002	0.062 ± 0.003	0.0002	family: Ruminococcaceae
360	0.063 ± 0.011	0.185 ± 0.016	0.013	family: Lachnospiraceae
362	0.705 ± 0.102	0.004 ± 0.000	0.007	family: Lachnospiraceae
413	0.110 ± 0.010	0.029 ± 0.011	0.004	family: Ruminococcaceae
415	0.021 ± 0.004	0.121 ± 0.011	0.002	genus: *Bacteroides*
417	3.263 ± 0.307	0.425 ± 0.109	0.002	family: Ruminococcaceae
431	0.053 ± 0.009	0 ± 0	0.012	genus: *Bacteroides*
436	0.155 ± 0.023	0 ± 0	0.007	family: Ruminococcaceae
449	0.187 ± 0.021	0 ± 0	0.001	genus: *Oscillospira*
459	3.334 ± 0.152	6.969 ± 0.549	0.010	genus: *Bacteroides*
470	0.025 ± 0.004	0.09 ± 0.007	0.002	genus: *Bacteroides*
477	0.049 ± 0.004	0 ± 0	0.0003	family: Lachnospiraceae
505	0.746 ± 0.108	0 ± 0	0.006	genus: *Subdoligranulum*
508	0.255 ± 0.024	0 ± 0	0.001	genus: *Bacteroides*
603	0.119 ± 0.011	0.002 ± 0.001	0.001	genus: *Clostridium*
605	0.260 ± 0.033	0.871 ± 0.063	0.002	genus: *Anaerotruncus*
608	0.016 ± 0.002	0 ± 0	0.006	family: Ruminococcaceae
657	2.281 ± 0.332	0 ± 0	0.007	genus: *Oscillospira*
703	0 ± 0	0.093 ± 0.013	0.006	famliy: ClostridialesFamilyXIII.IncertaeSedis
781	0.009 ± 0.001	0 ± 0	0.008	family: Ruminococcaceae
789	0.019 ± 0.002	0 ± 0	0.001	family: Lachnospiraceae
794	0.001 ± 0.000	0.007 ± 0.001	0.107	family: Lachnospiraceae
803	0.106 ± 0.012	0.016 ± 0.005	0.007	family: Ruminococcaceae
806	0.169 ± 0.015	0.645 ± 0.046	0.001	genus: *Bacteroides*
812	0.030 ± 0.003	0.006 ± 0.002	0.008	order: Clostridiales
814	0.005 ± 0.001	0 ± 0	0.128	Unclassified
834	0.006 ± 0.001	0.031 ± 0.004	0.008	genus: *Bacteroides*
852	0.038 ± 0.005	0 ± 0	0.003	genus: *Oscillospira*
853	0.079 ± 0.009	0 ± 0	0.002	family: Ruminococcaceae
875	0.400 ± 0.054	0.021 ± 0.004	0.006	genus: *Subdoligranulum*
933	0.013 ± 0.002	0.115 ± 0.013	0.004	genus: *Blautia*
945	0.141 ± 0.010	0 ± 0	0.0001	family: Ruminococcaceae

^a^Identified by SPLS-DA analysis.

^b^BD: basal diet; BG: BD diet supplemented with butyrate glycerides.

### Effect of dietary butyrate glycerides on *Bifidobacterium* and butyrate-producing bacteria

In the present study, genus *Bifidobacterium* was not detected in the digesta samples by the PCR primers universal to eubacterial 16S rRNA genes during pyrosequencing, which possibly resulted from a base-pair mismatch or low amplification efficiency of PCR primers as reported previously^18,19^. To investigate the effects of BG supplementation on the population of *Bifidobacterium*, a pair of PCR primers^20^ specific to the xylulose-5-phosphate⁄fructose-6-phosphate phosphoketolase (*xfp*) gene, which is characteristic of the *Bifidobacterium* genus^21^, were used for subsequent sequence analysis and qPCR assays. Approximately 5,000 bifidobacterial sequences per sample with an average length of 309 nucleotides were obtained from pyrosequencing. Among the sequences identified at the species level by comparison of the gene sequence similarities^21^, 46.9%, 49.9%, and 3.2% were assigned to *B. gallinarum*, *B. saeculare*, and *B. pullorum*, respectively, in the cecal samples from BG-treated birds. *B. saeculare* was the only *Bifidobacterium* species identified in the control group.

The effects of dietary treatment with BG on the relative abundance of *Bifidobacterium* and some groups of butyrate-producing bacteria were also assessed by qPCR assays. Supplementation of BG increased the *Bifidobacterium* abundance in the cecal samples by approximately 3 fold (Table 2). However, the supplementation had no effects (*P* > 0.05) on the abundance of Clostridial cluster IV and cluster XIVa that harbour butyrate-producing bacteria^22,23^. In addition, no significant difference (*P*>0.05) in the abundance of the gene encoding butyryl-CoA: acetate CoA transferase in the acetyl-CoA pathway, which is the most prevalent in bacterial production of butyrate^24^, was detected in the cecal samples between the control and treatment group.

**Table 2:** Fold changes in the abundance of selected cecal bacterial groups determined by qPCR.

Bacterium	Fold change (95% confidence interval, n = 6)		*P*-value
	BD^a^	BG	
Butyryl-CoA:acetate CoA transferase	1	1.19 (0.55–2.58)	0.6162
*Colostridial* cluster IV	1	1.06 (0.47–2.36)	0.8706
*Colostridial* cluster XIVa	1	1.38 (0.84–2.25)	0.1684
*Bifidobacterium* (genus)	1	2.84 (1.58–5.10)	0.0026

^a^BD: chickens on a basal diet. BG: chickens fed BD diet supplemented with butyrate glycerides.

### Effects of dietary butyrate glycerides on serum metabolites

**Figure 4:**
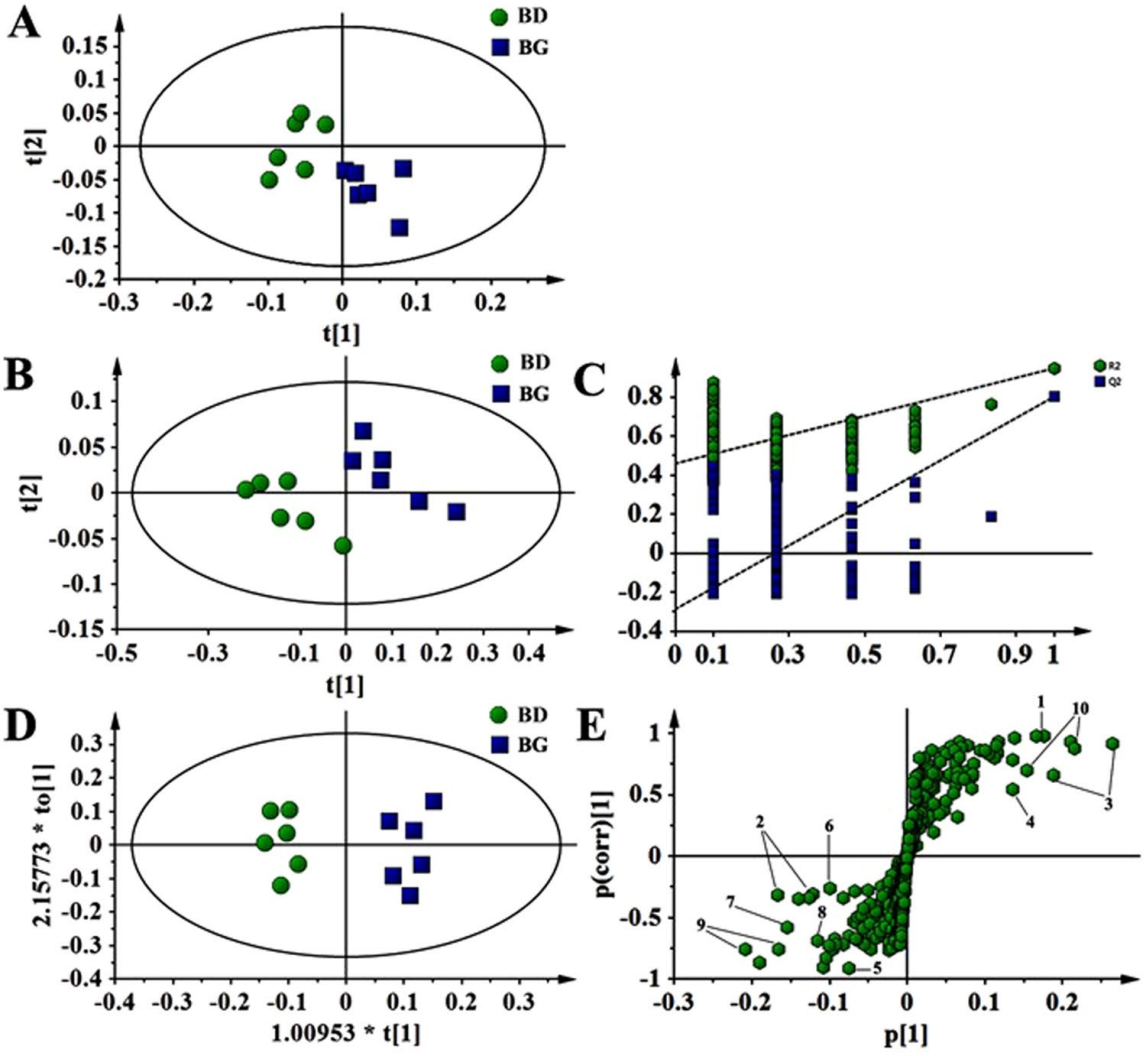
Pattern recognition with SIMCA-P 13.0. The PCA score plot (**A**), PLS-DA score plot (**B**), PLS-DA validation plots (permutation number: 200) (**C**), as well as OPLS-DA score plot (**D**) were derived from ^1^H NMR serum spectra of the BG-fed group compared with BD-fed chickens. Corresponding S-plot between BG-fed and BD-fed chickens, indicating the metabolites that changed significantly (panel E): 1, LDL/VLDL; 2, lipids; 3, lactate; 4, alanine; 5, succinate; 6, dimethylamine; 7, trimethylamine; 8, choline; 9, GPC; 10, TMAO.

A total of 42 metabolites were unambiguously identified by the ^1^H-NMR spectroscopic analysis in the samples from both treatments, and their chemical shifts, peak multiplicity, and the corresponding ^1^H NMR signal multiplicities were determined (Table 3). The assignment of metabolites was based on previous reports^25–27^. The spectra of the serum samples contained resonances from amino acids, organic acids, albumin, lipids, unsaturated lipids, choline, and creatine. Multivariate statistical analysis was used to detect subtle treatment-related metabolic differences^14,28^. Principal component analysis (PCA) was performed on the ^1^H NMR spectra of serum samples between BG-treated and untreated birds. The PCA score plot of the ^1^H NMR serum data is shown in Fig. 4A, with each point representing an individual spectrum of a sample showing separation of BG-treated birds from the untreated chicks. Partial least squares discrimination analysis (PLS-DA)-based profiling was also employed to explore the intrinsic differences between BG- and BD-fed groups. The samples from different groups were separated and classified into two distinct clusters as shown in the PLS-DA score plot (Fig. 4B). The model parameters (R^2^X = 0.563, R^2^Y = 0.932, Q^2^ = 0.608) and the validated model (permutation number: 200) indicated no over fitting (Fig. 4C). All of the results indicated the existence of differences between the two groups. Furthermore, the spectral datasets were analyzed by orthogonal partial least squares discriminant analysis (OPLS-DA). The BG-fed group was clearly separated from the BD-fed chickens as shown in the OPLS-DA scores plot (Fig. 4D) and by permutation tests and ANOVA of the cross-validated residuals (CV-ANOVA) (*P* ≤ 0.05). The metabolites responsible for a significant contribution to the separation of two groups are indicated in the corresponding S-plot, including low-density lipoprotein (LDL), very low-density lipoprotein (VLDL), lipids, lactate, alanine, succinate, dimethylamine, trimethylamine, choline, glycerophosphorylcholine (GPC), and trimethylamine-N-oxide (TMAO) that are numbered from 1 to 10 in Fig. 4E. The VIP statistic of the first principal component of OPLS-DA model (threshold ≥ 1), together with the *P*-value of the *t*-test (threshold ≤ 0.05), was used for selecting significant variables responsible for groups separation. Dietary supplementation of BG was accompanied by increased (*P* ≤ 0.05) concentrations of serum lactate, alanine, LDL/VLDL, and lipids. More interestingly, the concentrations of intestinal bacteria-derived metabolites also increased (*P* ≤ 0.05), including choline, GPC, dimethylamine, trimethylamine, TMAO and succinate (Table 4).

**Table 3:** Assignments of serum metabolites.

Key	Metabolite	Moiety	δ^1^H (ppm) & multiplicity
1	LDL/VLDL	CH_3_, CH_2_CH_2_C=	0.88(m), 1.28(m)
2	Isoleucine	γCH_3_, δCH_3_	0.94(t), 1.01(d)
3	Leucine	αCH, δCH_3_, δCH_3_	0.91(d), 0.96(d), 3.72(t)
4	Valine	αCH_3_, βCH, γCH_3_	0.99(d), 1.04(d)
5	Ethanol	CH_2_, CH_3_	3.65(q), 1.18(t)
6	β-Hydroxybutyrate	γCH_3_	1.22(d)
7	Lipids (triglycerides and fatty acids)	CH_3_(CH_2_)n, (CH_2_)n, CH_2_*CH_2_CO, CH_2_-C=C CH_2_-C=O, CH-O-CO	1.22 (m), 1.29 (m), 1.58(m), 2.04(m) 2.24(m), 2.75(m)
8	Threonine	αCH, βCH, γCH_3_	1.32(d), 4.25(m), 3.58(d)
9	Lactate	αCH, βCH_3_	1.33(d), 4.11(q)
10	Alanine	αCH, βCH_3_	3.77(q), 1.48(d)
11	Lysine	αCH, βCH_2_, γCH_2_, δCH_2_	3.77(t), 1.89(m), 1.73(m)
12	Acetate	CH_2_-C=O	1.92(s)
13	Glycoprotein	CH_3_-C=O	2.05(s), 2.08(m), 2.15(s)
14	Glutamate	αCH, βCH_2_, γCH_2_	3.75(m), 2.08(m), 2.37(m)
15	Pyruvate	CH_3_	2.37(s)
16	Succinate	α, βCH_2_	2.41(s)
17	Glutamine	αCH, βCH_2_, γCH_2_	3.68(t), 2.15(m), 2.45(m)
18	Methylamine	CH_3_	2.54(s)
19	Dimethylamine	CH_3_	2.71(s)
20	Trimethylamine	CH_3_	2.92(s)
21	Albumin	Lysyl-CH_2_	3.02(s)
22	Creatine	N-CH_3_, CH_2_	3.04(s), 3.93(s)
23	Creatinine	CH_3_, CH_2_	3.05 (s), 4.05(s)
24	Choline	N-(CH_3_)_3_, αCH_2_, βCH_2_	3.20(s), 4.05(t), 3.51(t)
25	GPC	N-(CH_3_)_3_, OCH_2_, NCH_2_	3.22(s), 4.32(t), 3.51(t)
26	TMAO	CH_3_	3.26(s)
27	Taurine	N-CH_2_, S-CH_2_	3.27(t), 3.43(t)
28	Betaine	CH_3_, CH_2_	3.28(s), 3.90(s)
29	Proline	βCH_2_, γCH_2_, δCH_2_	2.02–2.33(m), 2.00(m), 3.35(t)
30	Acetoacetate	CH_3_, CH_2_	2.29(s), 3.49(s)
31	Glycine	CH_2_	3.56(s)
32	Ornithine	CH_2_, αCH	3.80(s), 3.79(t)
33	Myo-Inositol	5-CH, 4, 6-CH, 2-CH	3.30(t), 3.63(t), 4.06(t)
34	β-Glucose	2-CH, 1-CH	3.25(dd), 4.65(d)
35	α-Glucose	1-CH	5.24(d)
36	Unsaturated lipids	= C-CH_2_-C= ,-CH=CH-	5.19(m), 5.31(m)
37	Fumarate	CH	6.52(s)
38	Tyrosine	αCH, CH_2_	7.19(d), 6.89(d)
39	Phenylalanine	2, 6-CH, 3, 5-CH, 4-CH	7.33(m), 7.38(m), 7.42(m)
40	1-Methylhistidine	4-CH, 2-CH	7.05(s), 7.77(s)
41	3-Methylhistidine	4-CH, 2-CH	7.00(s), 7.60(s)
42	Formate	CH	8.45(s)

Note: GPC, Glycerophosphorylcholine; LDL, Low-density lipoprotein; VLDL, Very low-density lipoprotein; TMAO, Trimethylamine N-oxide.

**Table 4:** Changes in relative concentrations of serum metabolites by butyrate glycerides.

No.	Metabolite	Chemical shift (δ)	FC^a^	VIP^b^	Change in direction	*P-*value^c^
1	LDL/VLDL	0.88	1.49	3.49	↑	0.013
2	Lipids	1.22	1.81	3.80	↑	0.030
3	Lactate	1.34	2.40	5.41	↑	0.002
4	Alanine	1.48	1.57	1.73	↑	0.046
5	Succinate	2.41	1.79	1.13	↑	0.040
6	Dimethylamine	2.71	2.03	1.31	↑	0.044
7	Trimethylamine	2.92	1.96	2.64	↑	0.035
8	Choline	3.20	2.53	1.30	↑	0.012
9	GPC	3.22	3.09	1.82	↑	0.006
10	TMAO	3.26	3.51	4.86	↑	0.002

Note: GPC, Glycerophosphorylcholine; LDL, Low-density lipoprotein; VLDL, Very low-density lipoprotein; TMAO, Trimethylamine N-oxide.

^a^FC: fold change between BG and BD diet-fed groups.

^b^Variable importance in the projection (VIP ≥ 1) was obtained from OPLS-DA analysis.

^c^*P*-value determined from Student’s *t*-test.

↑: a relative increase in the integral value for the region containing the identified metabolite.

## Discussion

The effects of butyrate on the intestinal microbiota of piglets and broiler chickens have been investigated previously by culture-based^29^ and non-culture-based methods, such as restriction fragment length polymorphism (RFLP) and fluorescence *in situ* hybridization (FISH)^30,31^. Due to the limitation of the methods, limited knowledge has been generated from these studies. The use of pyrosequencing in the present study uncovered a more comprehensive picture of the intestinal microbiota composition and structure. In the current study, dietary BG treatment did not affect the diversity of ileal and cecal microbiota, but altered the microbiota composition. This suggests that BG may have reached the lower intestinal tract like the caeca, owing to the hydrophilic nature of the monoglycerides in the form of monobutyrin. Compared with the change in the ileal microbiota (only *Bacillus*), there were several changes to the cecal bacteria, including to the class Mollicutes and genera *Eubacterium*, *Subdoligranulum, Holdemania*, *Anaerotruncus*, and *Bifidobacterium*. Many Mollicutes members cause diseases (including colitis) in humans and animals^32^. *Holdemania* has also been reported to be associated with unhealthy ceca and the use of antibiotics in animals^33,34^. The reduction in the abundance of Mollicutes and *Holdemania* in the present study suggests the beneficial effects of dietary BG treatment on chicken intestinal health. According to Bergey’s Manual of Systematic Bacteriology (the second edition), the genera *Subdoligranulum*^35^, *Eubacterium*^36^, and *Anaerotruncus*^37^ contain 1 (*Subdoligranulum variabile*), 44 (*Eubacterium*), and 1 (*Anaerotruncus colihominis*) species, respectively, among which many can produce butyric and lactic acids^22,38^. The decrease in the *Subdoligranulum* population by BG supplementation in the present study implies a functional suppression by butyrate released from BG, possibly as the result of feedback from the high level of butyrate in the chicken cecum. One striking observation in the present study is the effect of BG on the significant increase of *Bifidobacterium* in both diversity and abundance, as revealed by amplification of the *xfp* gene with both pyrosequencing analysis and qPCR assays. *Bifidobacterium* spp. are well-recognized probiotic bacteria with a wide spectrum of benefits^39^. Thus, the supplementation of BG likely promotes chicken health and well-being.

Recently, we reported the modulation of energy and lipid metabolism in young broiler chickens at the age of three weeks by dietary BG treatment^5^. There was up-regulation of genes involved in the biological processes for the reduction of synthesis, storage, transportation and secretion of lipids in the jejunum, and for the enhancement of the oxidation of ingested lipids and fatty acids in the liver. In particular, the gene expression of transcriptional regulators of thyroid hormone responsive (THRSP) and early growth response gene-1 (*EGR-1*) as well as some in the peroxisome proliferator-activated receptors (PPAR) signaling pathway was significantly affected by BG. Moreover, serum triglycerides and total cholesterol were lowered in BG-fed birds. While lipoprotein lipase was decreased in the jejunum, liver and adipose of BG-fed birds, fatty acid synthase levels were lowered in the serum, liver and adipose tissue of the same birds. In animals, energy and lipid metabolism is a complex process, which is regulated by a wide array of interdependent factors, including nutrients, hormones, nuclear transcription factors, and corresponding enzymes^40,41^. A change of the profiles of circulating metabolites may thus partially reflect the effects of dietary treatments on energy and lipid metabolism. There are two pathways, through which metabolites are involved in energy catabolism, including the pyruvate (alanine) production pathway and the “energy shift” pathway^42^. Alanine is most commonly produced by reductive amination of pyruvate with the consumption of ATP^43^; the higher serum concentration of alanine in BG-fed birds in the current study indirectly indicates an increase of pyruvate consumption as well as energy expenditure^44^. Another interesting observation is the relative higher concentrations of LDL/VLDL and lipids in the BG-fed birds than those in the control group, suggesting that BG promoted the transportation of lipids; thus reducing the storage of fatty acids in peripheral tissue^14,45^.

The intestinal microbiota can also affect host metabolism^46^. In the current study, dietary supplementation of BG increased the serum concentrations of choline, GPC, dimethylamine, trimethylamine, TMAO, and succinate when compared to those of control birds. These metabolites have been suggested to be derived from intestinal bacteria, particularly from *Bifidobacterium*, and are associated with several metabolic pathways^46^. For example, choline, dimethylamine, trimethylamine, and TMAO are mainly involved in the choline metabolic pathway to modulate lipid catabolism and glucose homeostasis^47–51^. It has been reported that major adverse cardiovascular events are normally accompanied with an increased level of TMAO^52^. It is unclear if the serum TMAO level observed in the present study was sufficient to trigger corresponding diseases in broilers. The GPC is a putative acetylcholine precursor that potentially increases growth hormone secretion and enhances fat oxidation^53^. Succinate is a key intermediate in microbial propionate synthesis^54^, and is also identified as a substrate for intestinal gluconeogenesis, a biological process that improves glucose homeostasis^55^. Collectively, enhanced serum concentrations of these metabolites by BG supplementation indicate that chicken intestinal bacteria can also contribute to lipid and energy metabolism through their metabolites to benefit the host.

Discovery of the relationships among the intestinal microbiota composition, microbiota-dependant metabolism, and animal performance can provide opportunities to improve food animal production. There have been studies on a link between bacterial species in the chicken intestine to the energy metabolism of broilers^56,57^. Some bacterial groups in the ileum and cecum^58,59^ or feces^60^ were related to feed conversion efficiency in chickens. For example, OTUs representing 26 bacterial species or phylotypes related to *Lactobacillus* spp., Ruminococcaceae, Clostridiales, Gammaproteobacteria, Bacteroidales, Clostridiales/Lachnospiraceae, and unclassified bacteria/clostridia in the ileum and cecum were found to be associated with feed efficiency of broiler chickens^58^. Recently, we reported that the supplementation of BG increased feeding efficiency by 10% and reduced abdominal fat deposition in 3-week-old broilers^5^. In the present study, 39 OTUs in the cecum of young birds were identified, which demonstrated changes in the relative abundance responding to BG treatment. Among these OTUs, Ruminococcaceae (18 OTUs) was the most predominant family followed by Bacteroidaceae (7 OTUs), Lachnospiraceae (6 OTUs), and order Clostridiales (5 OTUs). Only one OTU was assigned to *Lactobacillus*. These data support the previous observation by Torok *et al*.^58^. *Bifidobacterium* and *Faecalibacterium prausnitzii* are two groups of intestinal bacteria producing choline metabolites that can modulate lipid metabolism and glucose homeostasis^46^, and thus possibly alter animal health status and performance. The observations on the reduction of abdominal fat deposition in broilers in our previous study, and on the increase in serum concentrations of choline metabolites and in the diversity and abundance of *Bifidobacterium* in the chicken intestine by BG supplementation in the present study suggest that *Bifidobacterium* may have contributed to the decrease of abdominal fat deposition through the influence over the production of choline metabolites.

## Methods

### Animals, diets, and sample collection

The protocol for the animal trial was approved by the Animal Care and Use Committee of University of Guelph (AUP No. 3176) in accordance with the Canadian Council on Animal Care’s Guidelines. The feed ingredients and levels, including both BG additives and monensin, were the same as those used in the previous study [5]. The BG additives were mono-butyrin (mono-C4) and a mixture of 30% mono-, 50% di-, and 20% triglycerides of n-butyric acid (Baby C4), commercially available from SILO (Industria Zootecnica, Florence, Italy). No antibiotics were used throughout the trial except for monensin for the purpose of coccidiosis prevention.

Forty eight 1-d-old male birds (Ross 308; Stratford Chick Hatchery, Stratford, ON, Canada) were allocated equally into two dietary treatments: 1) basal diet-fed group (control), and 2) BG diet (basal diet + BG)-fed group. There were 24 birds per treatment with four birds each unit on the floor. The birds in the control group were fed a commercial diet for starter phase (0 to 20 d), while those in the BG diet-feed group consumed their assigned diet in a two phases program, namely a starter diet containing 3,000 ppm each of Mono C4 and Baby C4 from 0 to 7 d, and 3,000 ppm of Mono C4 only from 8 to 20 d. Feeding program, environmental temperature, and lighting schedules were the same as previously reported^5^. Feed and water were freely available.

All the birds appeared healthy and grew well throughout the entire experimental period. On d 20, six birds were randomly selected from each treatment (one bird per cage), and approximately 4 mL blood samples were collected from the jugular vein into 5 mL heparin-free vacutainer tubes (Becton Dickinson Vacutainer Systems, Franklin Lakes, NJ, USA). Samples were centrifuged at 750 g for 10 min at 4 °C, the supernatant (serum) was immediately collected and placed into test-tubes and stored at −20 °C until a NMR-based analysis. After blood collection, the same birds (six chickens per treatment) were killed by cervical dislocation, and digesta samples from the cecum and a 10-cm-long section of the ileum (5 cm away from the ileocecal junction) of each bird were aseptically collected, and stored at −80 °C until further analysis. There were 24 digesta samples collected in total with 12 samples from each treatment (6 birds per treatment with 2 segmental digesta samples of each bird).

### ^1^H-NMR spectroscopic analysis of serum

^1^H-NMR spectroscopic analysis of chicken serum samples was performed by the NMR Laboratory (nmr@chemistry.mcmaster.ca) in the Department of Chemistry and Chemical Biology, McMaster University (Hamilton, Ontario, Canada). One hundred microlitres of 0.9% saline in D_2_O with 1 mg/ml sodium [2,2,3,3-d4]3-trimethylsilyIpropanoate (TSP-d4) was mixed with 500 µl serum in high quality 5-mm NMR tubes. ^1^H NMR spectra of serum were recorded on a Bruker Avance III 700 MHz NMR spectrometer (Bruker Biospin, Rheinstetten, Germany) equipped with a 5 mm QNP cryo-probe and SampleJet autosampler, and operating at a proton frequency of 700.17 MHz. A carr-Purcel-Meiboom-Gill (CPMG) spin-echo pulse sequence [recycle delay−90°− (τ–180°–τ)_n_–acquisition] was used to emphasize resonances from low molecular-weight metabolites^14,28,61^. ^1^H NMR data for each sample were acquired using 128 scans (64k data points) with a 2.5 second relaxation delay. Chemical shifts were referenced to the internal reference (TSP-d_4_: 0.00 ppm). ^1^H NMR data with water suppression using excitation sculpting with gradients were subsequently acquired using the same number of scans and time of relaxation delay as that for ^1^H NMR data.

### Analysis of NMR data

The NMR spectra of serum samples were Fourier-transformed, phase adjusted, and baseline corrected using Mnova-6.1.1 (Mestrelab, Santiago de compostela, Spain) as previously described^14^. Chemical shifts were referenced to the internal reference (TSP-d_4_: 0.00 ppm). Each spectrum (δ 0.8–8.5) was segmented into contiguous segments having an equal width (0.01 ppm) and integrated over the region from equal width. The region δ 4.69–5.20 was removed to avoid the influence of the water signal. The integral of each region was determined. Resultant data sets were then imported into SIMCA-P 13.0 (Umetrics, Sweden) for multivariate statistical analysis. The PCA was applied to discern the presence of inherent similarities of spectral profiles and identify possible outliers within the dataset^62^. The PLS-DA was conducted to distinguish BG and BD diet-fed groups in a supervised manner. Parameters for model fitness (R^2^) and predictive ability (Q^2^) with leave-one-out cross validation and the response of the permutation test (200 times) needed to be used to evaluate whether the model could be established because of the small number of samples. Furthermore, a supervised pattern recognition approach known as OPLS-DA was used to improve the classification of the BG and BD diet-fed group while screening biomarkers. With an aim to discover the potential variables contributing to the differentiation, we generated an S-plot for the OPLS-DA model to define metabolites significantly contributing to the separation of the two groups. On the basis of the variable importance in the project (VIP) threshold of 1 (VIP ≥ 1.00), a number of metabolites responsible for the difference in metabolic profiles of the two groups could be obtained. In parallel, the metabolites identified by the OPLS-DA were validated at a univariate level using *t*-test (SPSS 17.0) with the critical *P*-value of 0.05 in order to detect the main metabolites that were significantly different in leading to the class discrimination^63^.

### Bacterial DNA extraction

Each digesta sample was processed individually for DNA extraction and each individual DNA sample was used for subsequent analyses of pyrosequencing and qPCR assays. Bacterial DNA was extracted using QIAamp DNA Stool Mini Kit (Qiagen, Valencia, CA, USA) in accordance with the manufacturer’s instructions. Briefly, approximate 0.2 g of digesta sample was lysed by incubation at 95 °C in Buffer ASL. PCR inhibitors and DNA-degrading substances were adsorbed to InhibitEX. Proteins were digested by incubation with Proteinase K at 70 °C. DNA was bound to the QIAamp silica-gel membrane and impurities were washed away. Purified DNA was eluted from the QIAamp spin column. The extracted bacterial DNA was stored at −80 °C until further analysis.

### DNA Pyrosequencing

Each individual digesta DNA sample was used to generate two PCR amplicon libraries that were combined for pyrosequencing. The PCR primers (universal to eubacteria) 28 F (5′-GAGTTTGATCNTGGCTCAG-3′) and 519 R (5′-GTNTTACNGCGGCKGCTG-3′) targeting the V1-V3 region of 16S rRNA genes were used to generate the amplicon libraries. Because of the insufficient sensitivity of eubacterial primers to *Bifidobacterium* 16S rRNA genes^18,19^, primers Bif-xfp-2294-F (5′-GAYGAGACCGCKTCCAACC-3′) and Bif-xfp-2691-R (5′-GAAGCCGTTGTGRTCCTGACG-3′), which target the 5-phosphate⁄fructose-6-phosphate phosphoketolase (*xfp*) gene of *Bifidobacterium*, were employed to produce amplicon libraries for pyrosequencing^20,21^. Pyrosequencing primers included the sequencing primer and an 8-nucleotide barcode. Amplicon libraries for the 16S rRNA gene and *xfp* gene were generated and analyzed separately. Bacterial tag-encoded FLX amplicon pyrosequencing was performed with Titanium on a Roche 454 FLX sequencer by the Research and Testing Laboratory in Lubbock (Texas, USA).

### Quantitative PCR assays

The qPCR assays were used to determine the relative abundance of genus *Bifidobacterium* and some bacterial groups relating to butyrate production, including Clostridial clusters IV and XIVa and bacteria harboring the gene of butyryl-CoA:acetate CoA transferase^22^. The assays were performed with individual digesta DNA samples separately using a S1000 Thermocycler (1852148, Bio-Rad Laboratories, Hercules, CA, USA). The following was the thermal cycle profile: 95 °C for 3 min; 40 cycles of 95 °C for 15 sec, a specific annealing temperature for 30 sec, and 72 °C for 30 sec. A thermal melt curve was generated by heating at 95 °C for 1 min, 55 °C for 30 sec, and ramping back to 95 °C in 0.5 °C increments. The 25-μl reaction mixture contained 1.0 μl of DNA template, 12.5 μl of 2× iTaq SYBR Green Supermix (Bio-Rad, Hercules, CA, USA), and forward and reverse primers (Table S1). The abundance of the different groups of bacteria was normalized using the amplicon from the universal PCR primers towards eubacteria^64^, which served as an internal housekeeping control. Fold changes in the abundance of different group bacteria were calculated by the 2^−∆∆Ct^ method^65^. The PCR primers were those used for *Bifidobacterium*^20^, Butyryl-CoA:acetate CoA transferase^66^, Colostridial cluster IV^67^, and Colostridial cluster XIVa^68,69^, respectively (Table S1).

### Bioinformatics and statistical analysis of microbiota

The data generated from each sample (either pyrosequencing or qPCR assays) were analyzed separately before combination for the analysis of treatment effects. Raw sequence data were denoised using the software USEARCH^70^. Detection and removal of chimeras were performed using the software UCHIIME^71^. Sequences with low quality and less than 250 bp in length were removed. Sequences passing the quality control screening were processed by the Quantitative Insights Into Microbial Ecology (QIIME) pipeline (http://qiime.sourceforge.net). Barcode and primers were removed during the demultiplexing step. The sequences were grouped into OTUs at least 97% similarity using UCLUST. For the 16S rRNA gene, a representative sequence was picked from each OTU and assigned taxonomy using the Ribosomal Database Project (RDP) naïve Bayesian classifier^72^ with the confidence threshold as 80%. The sequences were aligned against the Greengenes-imputed core reference alignment using PyNAST, and the concatenated alignment of OTU was filtered to remove gaps and hypervariable regions using the Greengenes Lane mask. The filtered sequences alignment was then used to build a phylogenetic tree for calculation of UniFrac diversity. Data were rarefied to the lowest counts of sequences per sample for calculation of alpha and beta diversities. The PCoA based on the unweighted UniFrac distance was implemented in the software QIIME. For the sequences of the *xfp* gene, taxonomic assignments of the OTUs were made by comparison to a reference database of the *xfp* gene^21,73^. The ANOSIM provides a way to test whether similarities within groups are higher than those between groups, thus allowing for testing whether bacterial communities were different between two or more groups^74^. This nonparametric test was implemented in R package vegan (version 2.0–4), and UniFrac distance was used as a measure of dissimilarity of bacterial communities in the ileal or cecal digesta^75^. When the ANOSIM test indicated a difference of bacterial communities between the control and BG-treated group, SPLS-DA was utilized to select OTUs that could be used for separation of the two groups using the R package spls^76,77^. Sparse partial least squares regression can achieve variable selection and dimension reduction simultaneously. For our data, the binary response was the dietary treatment, *i.e*. 6 birds were selected from each of the control and BG-treated group. The relative abundance of OTUs was normalized as percentage of total number of sequences for an individual bird and related to the corresponding dietary treatment. A heat map of the relative abundance of selected OTUs in the cecal digesta was created by the R package gplots. Relative abundances of taxonomy and OTU were compared between the control and BG-treated group using a permutation test via online software Metastats^17^. The qPCR data were subjected to simple t-test^78^. Significance level was set at 0.05.

## Electronic supplementary material

Supplementary Information
