# Supplementary material for: Dietary butyrate glycerides modulate intestinal microbiota composition and serum metabolites in broilers
Source: Sci Rep. 2018 Mar 21;8:4940. doi: 10.1038/s41598-018-22565-6 (PMC5862971; doi:10.1038/s41598-018-22565-6)
Supplement: Supplementary file 1 — Supplementary Information [file 41598_2018_22565_MOESM1_ESM.docx]

**Dietary butyrate glycerides modulate intestinal microbiota composition and serum metabolites in broilers**

Xiaojian Yang^1§^, Fugui Yin^1,2§^, Yuhui Yang^3^, Dion Lepp^1^, Hai Yu^1^, Zheng Ruan^4^, Chengbo Yang^5^, Yulong Yin^2,4,6^, Yongqing Hou^6^, Steve Leeson^7^, Joshua Gong^1*^

**Figure S1. Effects of butyrate glycerides on the α-diversity of ileal (A) and cecal (B) microbiota.** Alpha rarefaction curves for the observed number of OTUs, Chao1 index, Shannon index, and phylogenetic diversity. Values are means ± standard deviations. BD: basal diet; BG: BD supplemented with butyrate glycerides.


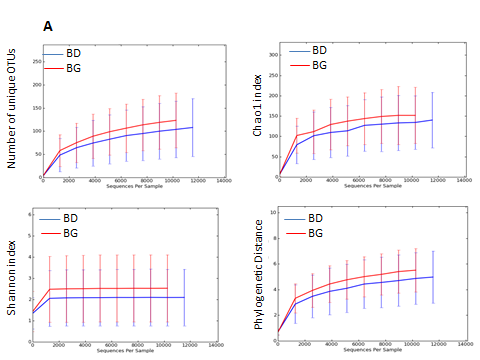


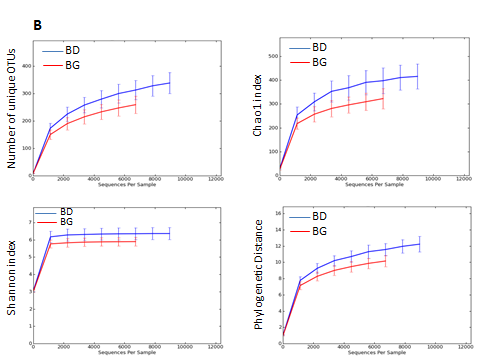


**Figure S2. Effects of butyrate glycerides on the microbiota composition in the ileum and cecum of 20-day-old broilers.** The bar charts represent the relative abundance (%) of bacterial phyla (A) and families/genera (B) in the ileal and cecal microbiota of chickens (≥ 0.005% of sequences). Classification is determined according to the RDP trained on the Greengenes database with a minimum confidence score of 0.8. N = 6. BD: basal diet; BG: BD supplemented with butyrate glycerides.

**A**


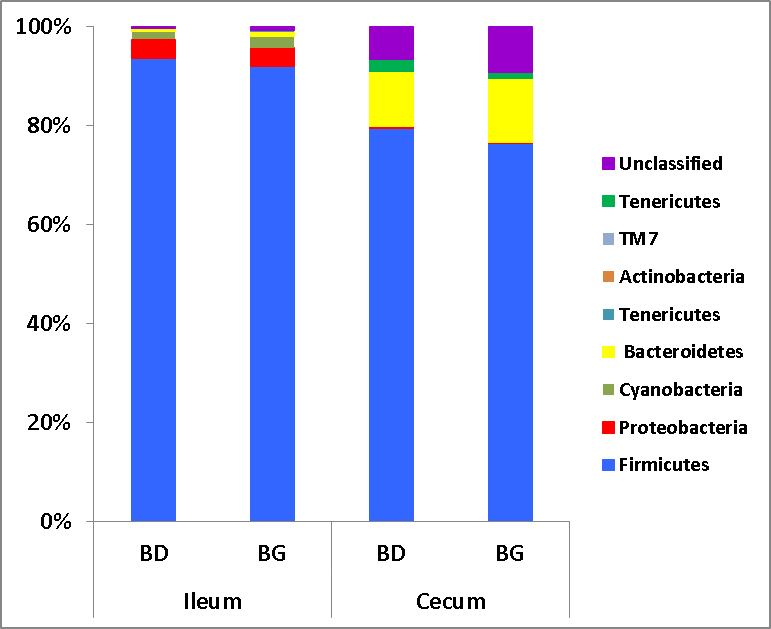


**B**


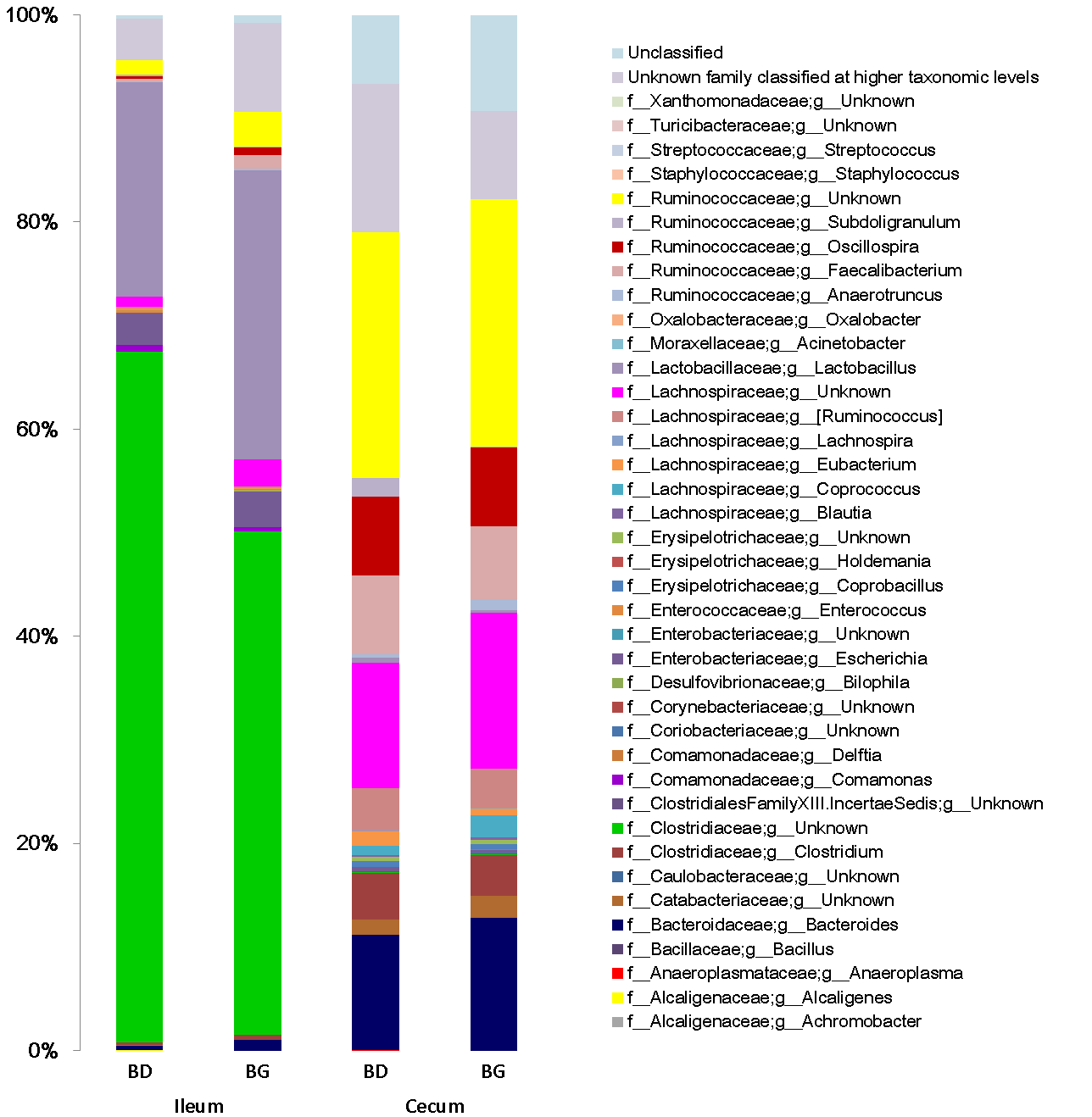


**Table S1.** PCR primers

| Targeted bacterial group | Amplicon size (bp) | Annealing  temperature (°C) | Primer (5’-3’) |
| --- | --- | --- | --- |
| Total eubacteria | 200 | 58 | Forward: ACTCCTACGGGAGGCAGCAG  Reverse: GTATTACCGCGGCTGCTGGCAC |
| Butyryl-CoA:acetate CoA transferase | 530 | 53 | Forward: GCIGAICATTTCACITGGAAYWSITGGCAYATG  Reverse: CCTGCCTTTGCAATRTCIACRAANGC |
| Colostridial cluster IV | 239 | 50 | Forward: GCACAAGCAGTGGAGT  Reverse: CTTCCTCCGTTTTGTCAA |
| Colostridial cluster XIVa | 438-441 | 50 | Forward: AAATGACGGTACCTGACTAA  Reverse: CTTTGAGTTTCATTCTTGCGAA |
| *Bifidobacterium* (genus) | 300 | 58 | Forward: GAYGAGACCGCKTCCAACC  Reverse: GAAGCCGTTGTGRTCCTGACG |
